# Integration of genome-wide association study and expression quantitative trait locus mapping for identification of endometriosis-associated genes

**DOI:** 10.1038/s41598-020-79515-4

**Published:** 2021-01-12

**Authors:** Ya-Ching Chou, Ming-Jer Chen, Pi-Hua Chen, Ching-Wen Chang, Mu-Hsien Yu, Yi-Jen Chen, Eing-Mei Tsai, Shih-Feng Tsai, Wun-Syuan Kuo, Chii-Ruey Tzeng

**Affiliations:** 1 Department of Biological Science and Technology, College of Biological Science and Technology, National Chiao Tung University, Hsinchu, Taiwan; 2 Center for Intelligent Drug Systems and Smart Bio-devices (IDS2B), National Chiao Tung University, Hsinchu, Taiwan; 3 Center for Reproductive Medicine and Sciences, Department of Obstetrics and Gynecology, Taipei Medical University Hospital, Taipei, Taiwan; 4 Department of Obstetrics and Gynecology, School of Medicine, College of Medicine, Taipei Medical University, Taipei, Taiwan; 5 Department of Obstetrics and Gynecology and Women’s Health, Taichung Veterans General Hospital, Taichung, Taiwan; 6 School of Medicine, National Yang-Ming University, Taipei, Taiwan; 7 Graduate Institute of Clinical Medicine, College of Medicine, Taipei Medical University, Taipei, Taiwan; 8 Department of Obstetrics and Gynecology, Shuang Ho Hospital, Taipei Medical University, Taipei, Taiwan; 9 Department of Obstetrics and Gynecology, Tri-Service General Hospital, National Defense Medical Center, Taipei, Taiwan; 10 Department of Obstetrics and Gynecology, Taipei Veterans General Hospital, Taipei, Taiwan; 11 School of Medicine, Institute of Clinical Medicine, National Yang-Ming University, Taipei, Taiwan; 12 General Research Centers of R&D Office, Kaohsiung Medical University, Kaohsiung, Taiwan; 13 Division of Reproductive Medicine, Department of Obstetrics and Gynecology, Kaohsiung Medical University Hospital, Kaohsiung, Taiwan; 14 Institute of Molecular and Genomic Medicine, National Health Research Institutes, Miaoli, Taiwan

**Keywords:** Genetic association study, Reproductive disorders

## Abstract

To determine whether genetic predisposition to endometriosis varies depending on ethnicity and in association with expression quantitative trait loci (eQTL) in a Taiwanese population. We conducted a genome-wide association study (GWAS) and replicated it in 259 individuals with laparoscopy-confirmed stage III or IV endometriosis (cases) and 171 women without endometriosis (controls). Their genomic DNA was extracted from blood and evaluated by the GWAS of Taiwan Biobank Array. Novel genetic variants that predispose individuals to endometriosis were identified using GWAS and replication, including rs10739199 (*P* = 6.75 × 10^−5^) and rs2025392 (*P* = 8.01 × 10^−5^) at chromosome 9, rs1998998 (*P* = 6.5 × 10^−6^) at chromosome 14, and rs6576560 (*P* = 9.7 × 10^−6^) at chromosome 15. After imputation, strong signals were exhibited by rs10822312 (*P* = 1.80 × 10^−7^) at chromosome 10, rs58991632 (*P* = 1.92 × 10^−6^) and rs2273422 (*P* = 2.42 × 10^−6^) at chromosome 20, and rs12566078 (*P* = 2.5 × 10^−6^) at chromosome 1. We used the Genotype-Tissue Expression (GTEx) database to observe eQTL. Among these SNPs, the cis-eQTL rs13126673 of inturned planar cell polarity protein (INTU) showed significant association with INTU expression (*P* = 5.1 × 10^–33^). Moreover, the eQTL analysis was performed on endometriotic tissues from women with endometriosis. The expression of INTU in 78 endometriotic tissue of women with endometriosis is associated with rs13126673 genotype (*P* = 0.034). To our knowledge, this is the first GWAS to link endometriosis and eQTL in a Taiwanese population.

## Introduction

Endometriosis affects 6–10% of women of reproductive age and is a common gynecological disorder, with 20–50% of women with endometriosis experiencing infertility^1^. The disease is characterized by the presence of endometrial tissue outside the uterus and is highly associated with pelvic pain. The pathogenesis of endometriosis is hypothesized to result from fragment of the endometrium moving back, implanting on peritoneal surfaces and continuing to grow^2^. However, this implantation hypothesis is not well accepted. Family history of endometriosis has been reported to increase relative risk about five-fold^3,4^. From twin studies, the total heritability of endometriosis is 0.47–0.51 with a common SNP-based heritability of 0.26^1,5,6^.

Until now, several genome-wide association studies (GWAS) have found many independent single-nucleotide polymorphisms (SNPs) associated with endometriosis. The first GWAS was conducted by Uno et al. and found SNP rs10965236 located in CDKN2BAS on the chromosome 9p21 to be associated with endometriosis in a Japanese population^7^. The second study identified SNP rs12700667 on chromosome 7p15.2 to be associated with endometriosis in a European cohort^8^. Meta-analysis also identified important loci associated with endometriosis and highlighted the genes involved in hormone metabolism, which is the largest endometriosis GWAS published to date (over 17,000 cases and 191,000 controls)^9,10^. It is important to identify the genetic factors of endometriosis in different ethnicities. Recently, a pooling-based genome-wide scan was conducted and demonstrated ten ovarian endometrioma-associated loci^11^.

Herein, the GWAS are performed in the Taiwanese population to find novel variants of endometriosis. After imputation, strong signals were exhibited. Moreover, the expression quantitative trait loci (eQTL) explain the variation in expression levels of mRNA; thus, in this study, we used the Genotype-Tissue Expression (GTEx) database and detected the cis-eQTL in our endometriotic tissues. Thus, we aimed to clarify novel susceptibility loci of endometriosis and the eQTL within a Taiwanese population.

## Results

### Assessment of population stratification

**Figure 1:**
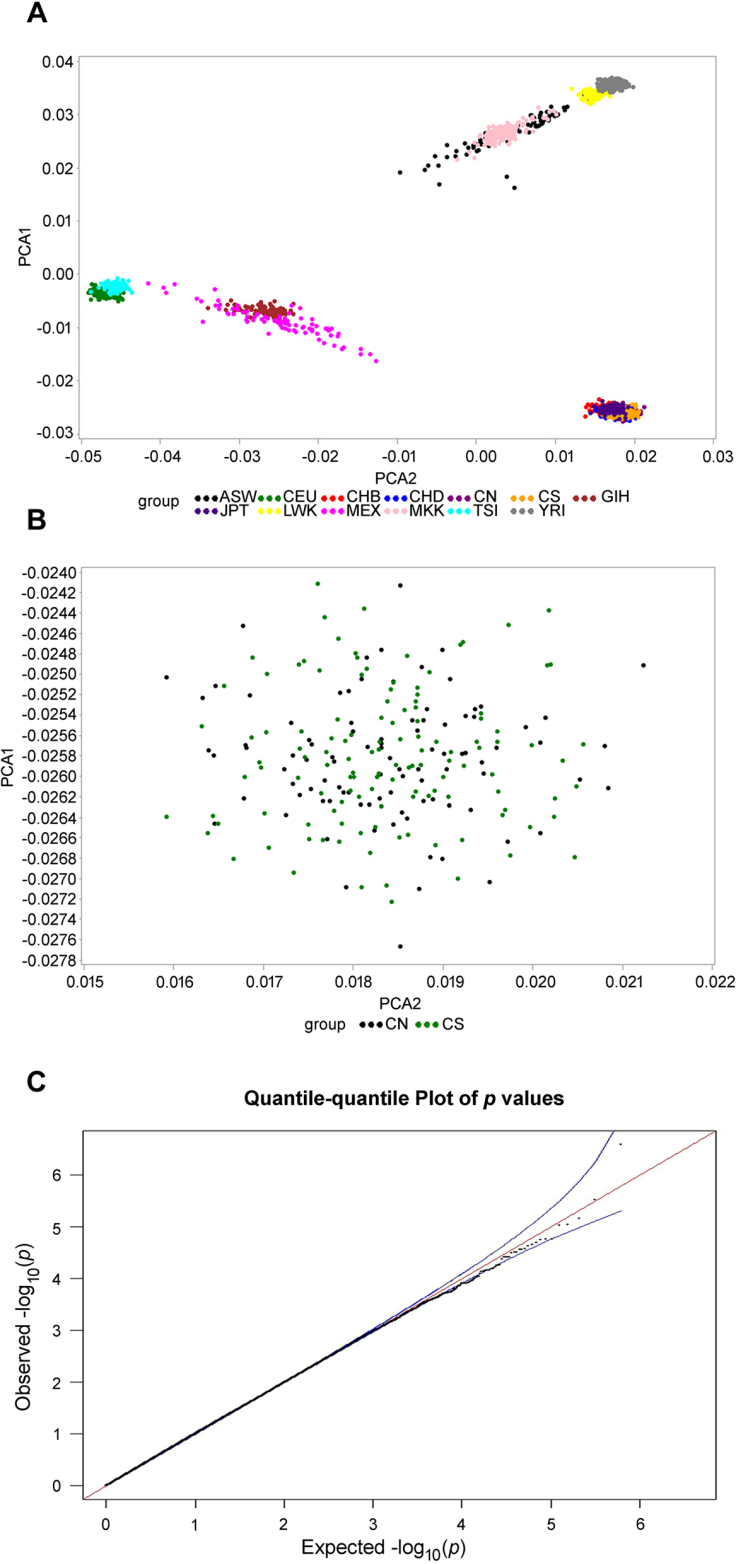
Multidimensional scaling analysis. (**A**) The results of multidimensional scaling analysis of the GWAS samples with HapMap populations, represented with principal component analysis (PCA) plot. (**B**) The results of multidimensional scaling analysis of the GWAS sample only. (**C**) Quantile (Q)–Quantile (Q) plot of the *P* value in Cochran–Armitage trend test. The lambda (λ) value is 1.01. *CA* case, *CN* control, *GWAS* genome-wide association study.

The demographic results are shown in Table 1. We performed a case–control GWAS to identify loci associated with increased risk of endometriosis in the Taiwanese population using an Affymetrix Axiom TWB array containing 653,291 SNP probes. We initially enrolled 126 endometriosis and 96 non-endometriosis controls from a Taiwanese population residing in Taiwan. After kinship analysis and strict quality control filtering, we analyzed 620,465 SNPs representing 95% of the array SNPs for the samples from the GWAS group. Multidimensional scaling analysis and results of permutation tests for identity-by-state revealed no differences between the endometriosis and control groups, providing no evidence for strong population stratification (Fig. 1A,B). Quantile–quantile (Q–Q) plots were used to examine *P* value distributions, and the lambda value (λ) was 1.01 on the basis of the *P* value from the Cochrane–Armitage trend test, indicating no population substructure (Fig. 1C). In total, we found 33 top SNPs associated with endometriosis (*P* < 1 × 10^−4^) (Supplementary Table 1).

**Table 1:** Baseline demographic summary of women with endometriosis and control (n = 430).

Characteristics	Discovery study			Replication study		
	Case n = 126 (%)	Control n = 96 (%)	*P* value	Case n = 133 (%)	Control n = 75 (%)	*P* value
Age, years^a^	35.49 (6.42)	37.78 (6.96)	0.0118	36.76 (7.15)	41.39 (6.57)	< 0.0001
BMI^a^, kg/m^2^	21.06 (2.79)	23.13 (4.50)	< 0.0001	21.8 (3.5)	23.97 (4.77)	0.0008
Age of menarche^a^	12.66 (1.32)	12.58 (1.28)	0.8739	13.01 (1.32)	12.63 (1.8)	0.048
Duration of menstrual cycle^a^	28.06 (2.41)	28.43 (4.52)	0.5325	28.62 (3.29)	27.14 (4.25)	0.0227
Dysmenorrhea, n (%)^b^	103 (81.75)	57 (59.38)	0.0002	99 (74.44)	58 (77.33)	0.6409

Mean (SD) for continuous variables.

n (%) for discontinuous variables.

*BMI* body mass index, *SD* standard deviation.

^a^Mann–Whitney test.

^b^χ^2^ test.

### GWAS and cross-platform validation

**Figure 2:**
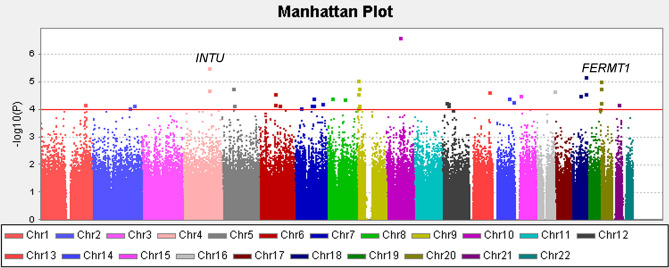
Manhattan plot of endometriosis. Results of genome-wide association analysis (− log_10_ *P*) presented in chromosomal order for 620,465 SNPs tested for association in 126 endometriosis and 96 non-endometriosis control. The *x* axis shows each of the SNPs used in the discovery phase. The *y* axis shows the − log_10_ *P* value of the trend test. Signals in inturned planar cell polarity protein (*INTU*) and fermitin family member 1 (*FERMT1*) loci are also presented. *SNP* single nucleotide polymorphism.

The GWAS analysis was conducted with 126 endometriosis cases and 96 controls. The Manhattan plot showed the result of genome-wide association analysis (−log_10_ *P*) in chromosomal order for 620,465 SNPs test (Fig. 2). A minimum of 99% calling of Affymetric in both endometriosis cases and controls was selected for cross-platform validation using a Sequenom MassARRAY (Supplementary Table 1).

### Replication of top variants for endometriosis

**Figure 3:**
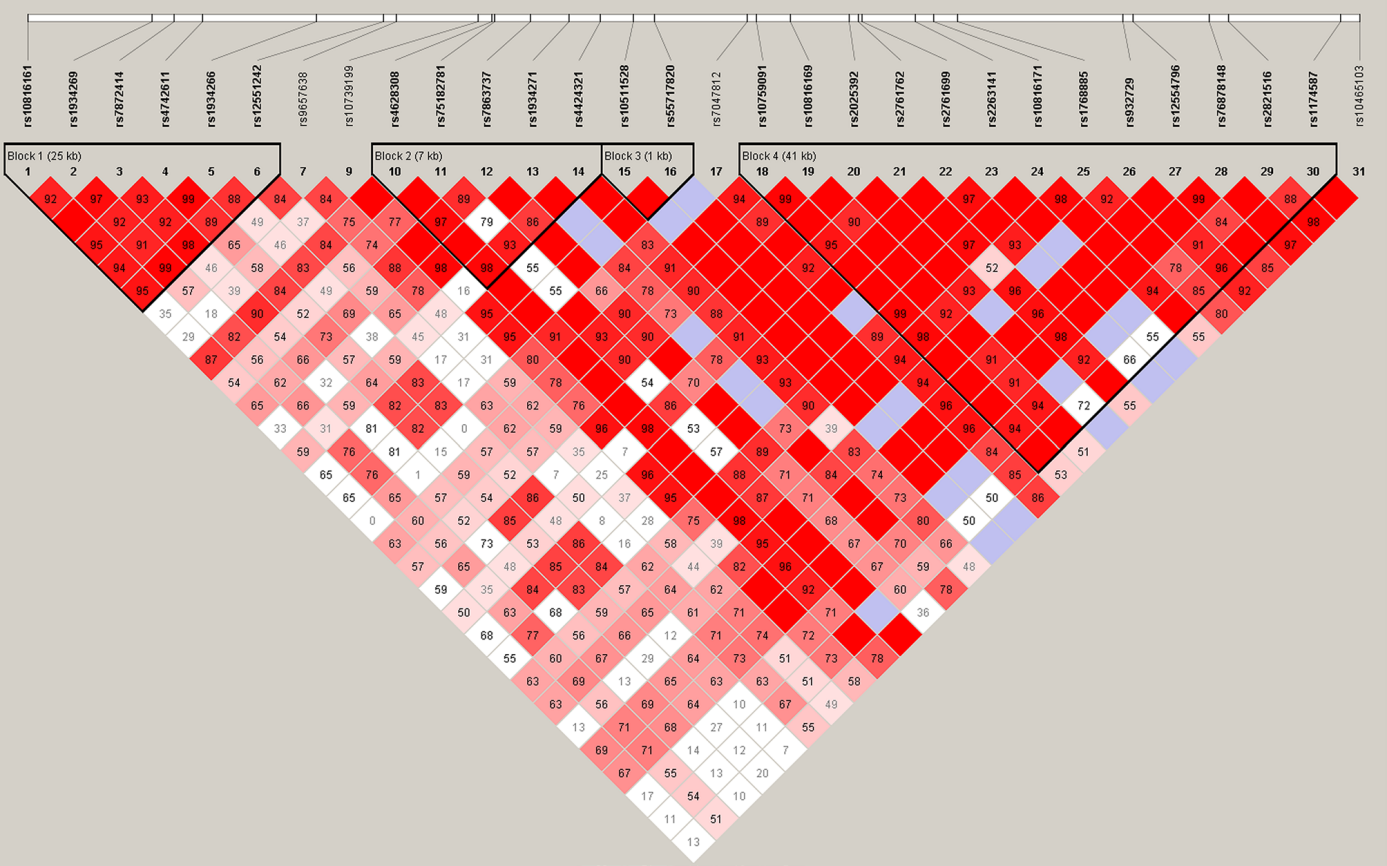
Linkage disequilibrium (LD) plot for the 31 *PTPRD* SNPs analyzed. The values in boxes are pair-wise SNP correlations (D′); bright red boxes without numbers indicate complete LD (D′ = 1). The texts above the horizontal numbers are the SNP names, and the blocks indicate haplotype blocks.

The 33 SNPs (Supplementary Table 1) were tested in the replication stage and an independent cohort of 133 patients with endometriosis and 75 controls using a Sequenom MassARRAY and Q-PCR (Supplementary Table 2). After multiple test analyses using Bonferroni correction, the association was found to be not significant. In a combined analysis of the GWAS and replication cohorts, *P* values for 4 of the identified SNPs were lower than 10^−4^, which were not genome-wide significant (*P* < 5.0 × 10^–8^) (Table 2). We found that the SNPs rs10739199 (*P* = 6.75 × 10^−5^) and rs2025392 (*P* = 8.01 × 10^–5^) located at chromosome 9 in *PTPRD* (protein tyrosine phosphatase, receptor type D). Two SNPs (rs10739199 and rs2025392) were found in linkage disequilibrium (LD; D’ = 0.961 and r^2^ = 0.208, Fig. 3). After GWAS conditional analyses of these two SNPs, the *P* values were increased and indicated that they were only both associated. The *P* values for other 2 SNPs were lower than 10^–5^. These two SNPs were located at chromosome 14 (rs1998998, *P* = 6.5 × 10^−6^) and at chromosome 15 (rs6576560, *P* = 9.7 × 10^−6^). These were all replicated in the independent population and calculated in joint analysis (Table 2). The *P* value of joint analysis is shown in Table 3 and Supplementary Table 3. However, the *P* value did not reach the standard genome-wide threshold (*P* value lower than 5 × 10^–8^).

**Table 2:** SNPs with *P* values < 1 × 10^–4^ in joint analysis.

Chr	SNP	Position	Gene	Allele format	Risk allele	Stage	Control/case	RAF controls	RAF cases	Trend P	OR	95% CI	
9	rs10739199	9,707,144	PTPRD	GA	A	GWAS	96/126	0.6094	0.8016	1.70E−05	2.59	1.695	3.958
				GA		Replication	75/133	0.6486	0.7154	1.73E−01	1.361	0.8841	2.097
				GA		Combined	171/259	0.6265	0.7578	6.75E−05	1.866	1.384	2.515
9	rs2025392	9,733,309	PTPRD	TC	C	GWAS	96/126	0.8542	0.964	7.30E−05	4.572	2.103	9.941
				TC		Replication	75/133	0.9189	0.947	2.45E−01	1.576	0.7088	3.503
				TC		Combined	171/259	0.8824	0.9553	8.01E−05	2.846	1.671	4.848
14	rs1998998	97,680,819	–	AG	A	GWAS	96/126	0.1146	0.268	5.31E−05	2.829	1.674	4.782
				AG		Replication	75/133	0.1486	0.2386	2.38E−02	1.795	1.052	3.062
				AG		Combined	171/259	0.1294	0.2529	6.50E−06	2.277	1.567	3.31
15	rs6576560	26,577,347	–	TC	C	GWAS	96/126	0.4896	0.6905	3.09E−05	2.326	1.576	3.432
				TC		Replication	75/133	0.527	0.6364	3.78E−02	1.571	1.044	2.363
				TC		Combined	171/259	0.5059	0.6628	9.70E−06	1.92	1.451	2.541

*RAF* risk allele frequency, *Trend P* *P* vale of Trend test, *OR* odds ratio, *CI* confidence interval.

**Table 3:** SNPs with p values < 1 × 10^–3^ in Joint analysis.

Chr	SNP	Position	Gene	Allele 1	Allele 2	Risk allele	RAF controls	RAF cases	Joint Trend P	OR	95% CI		HWE test P
4	rs13126673	128,586,241	INTU	T	C	C	0.4793	0.6142	1.10E−04	1.729	1.309	2.284	0.0209
5	rs2056401	52,363,759	ITGA2	G	A	A	0.7735	0.8643	4.76E−04	1.865	1.304	2.668	0.5196
7	rs7789771	90,650,256	CDK14	G	A	G	0.04734	0.1152	7.21E−04	2.621	1.481	4.638	0.0423
7	rs1358101	90,660,684	CDK14	A	C	A	0.04706	0.1148	7.03E−04	2.626	1.484	4.646	0.0418
9	rs10739199	9,707,144	PTPRD	G	A	A	0.6265	0.7578	6.75E−05	1.866	1.384	2.515	0.7429
9	rs2025392	9,733,309	PTPRD	T	C	C	0.8824	0.9553	8.01E−05	2.846	1.671	4.848	0.7065
9	rs2761699	9,734,220	PTPRD	C	T	T	0.8853	0.9512	4.05E−04	2.524	1.497	4.255	1
10	rs10822312	66,314,543	–	T	G	G	0.6568	0.7724	4.36E−04	1.773	1.307	2.405	0.0407
14	rs10136321	75,785,259	–	A	T	A	0.4294	0.5449	9.59E−04	1.591	1.207	2.098	0.8758
14	rs1998998	97,680,819	–	A	G	A	0.1294	0.2529	6.50E−06	2.277	1.567	3.31	1
15	rs6576560	26,577,347	–	T	C	C	0.5059	0.6628	9.70E−06	1.92	1.451	2.541	0.6472
18	rs1870631	41,362,595	–	A	G	G	0.4735	0.5872	8.31E−04	1.582	1.2	2.084	0.8776

*RAF* risk allele frequency, *Trend P* *P* vale of Trend test, *OR* odds ratio, *CI* confidence interval, *HWE test P* Hardy–Weinberg equilibrium test *P* value of controls.

**Figure 4:**
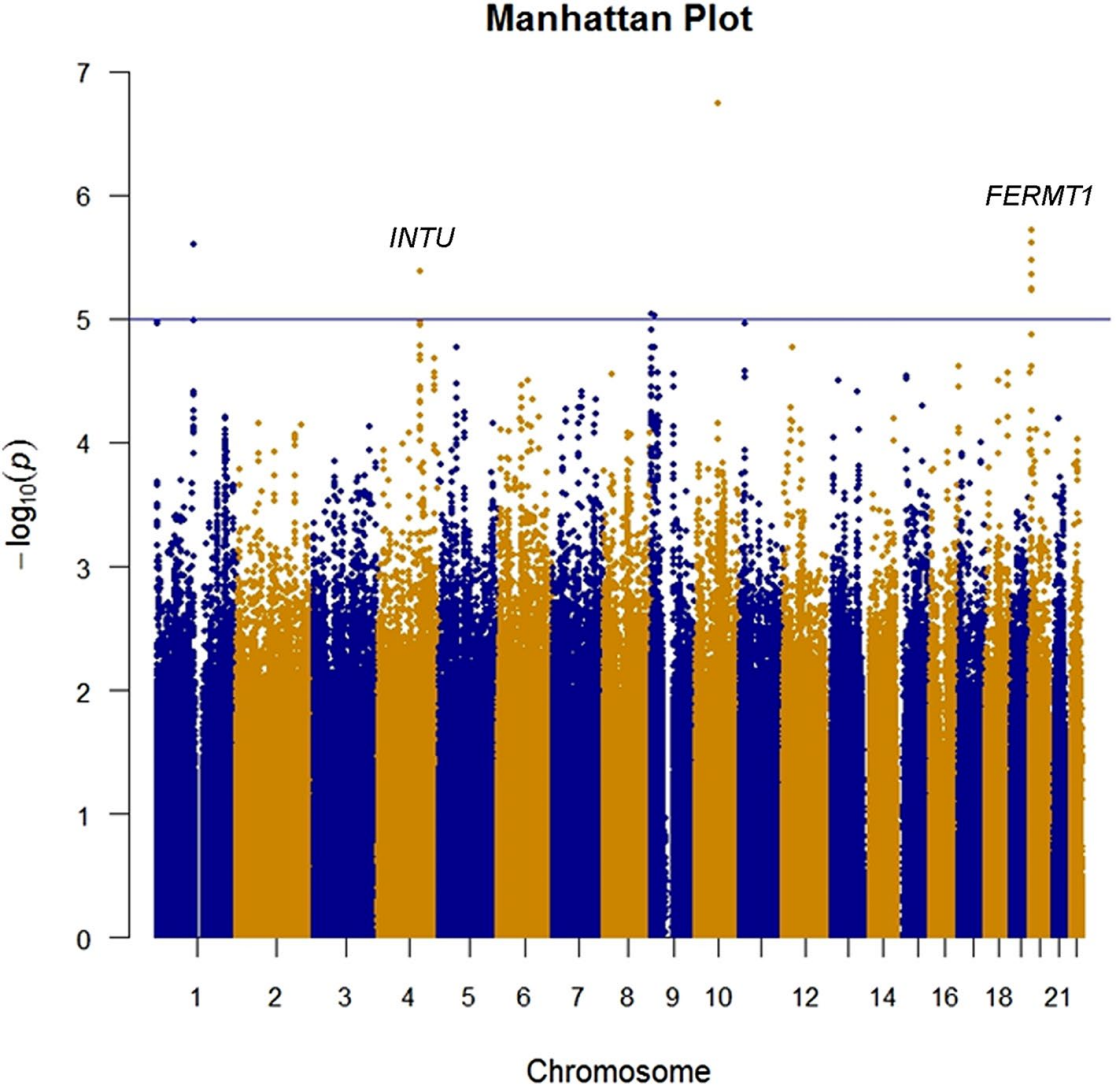
Imputation of GWAS. Results of genome-wide association analysis (− log_10_ *P*) presented in chromosomal order for 4,566,885 SNPs imputed for association between 126 endometriosis and 96 non-endometriosis control. The *x* axis shows each of the SNPs used in the discovery phase. The *y* axis shows the − log_10_ *P* value of the trend test. Signals in inturned planar cell polarity protein (*INTU*) and fermitin family member 1 (*FERMT1*) loci are presented. *SNP* single nucleotide polymorphism.

**Figure 5:**
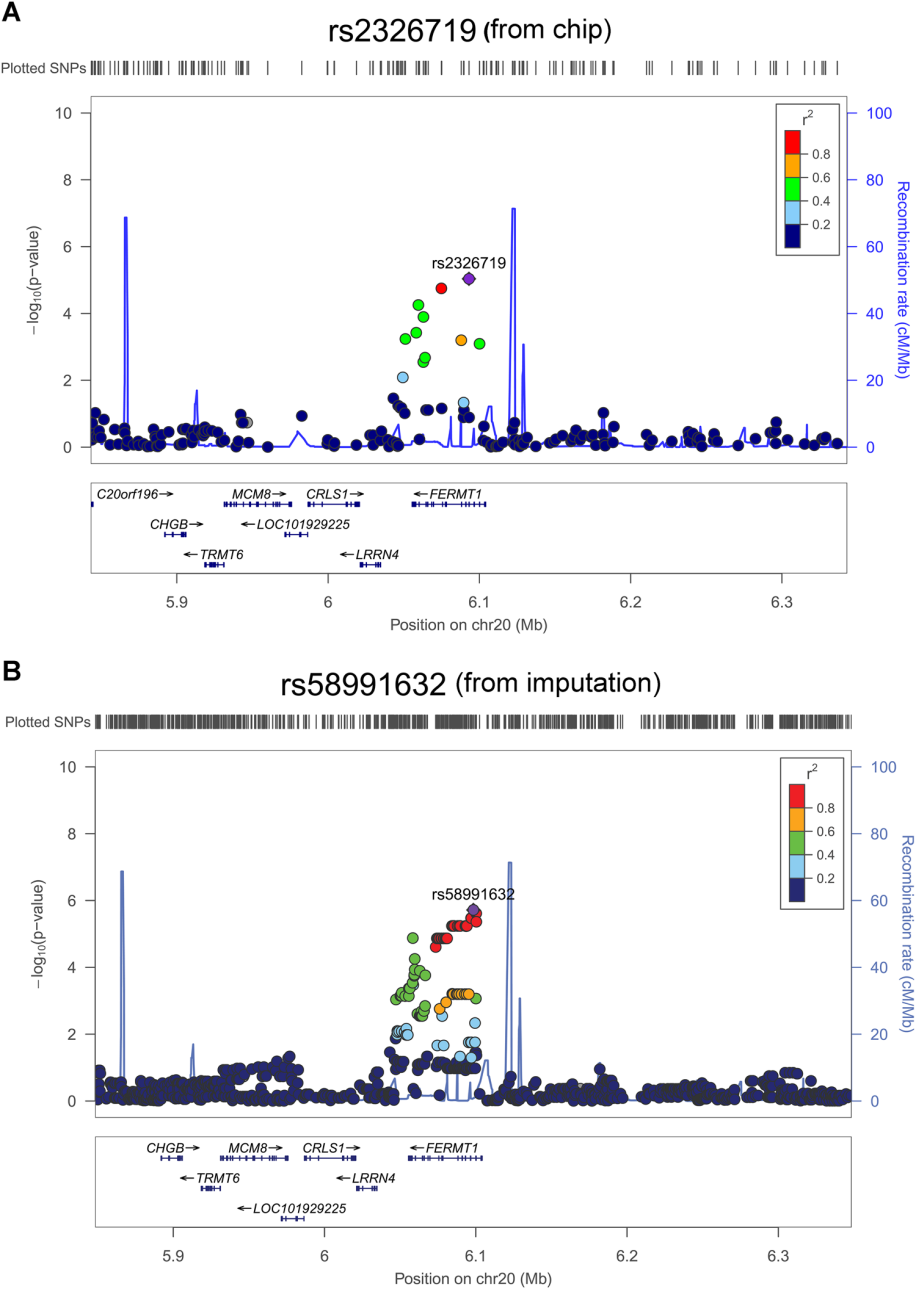
Association plots for the *FERMT1* locus. (**A**) Reginal association plot for the *FERMT1* locus on chromosome 20 with gene annotation imposed. Each SNP was plotted to represent its chromosomal location (x-axis) and − log10 *P* value (y-axis) for the trend test from the GWAS data. (**B**) After imputation, the SNPs were plotted, and the colors denote the strength of linkage disequilibrium of SNPs to *FERMT1.*

**Figure 6:**
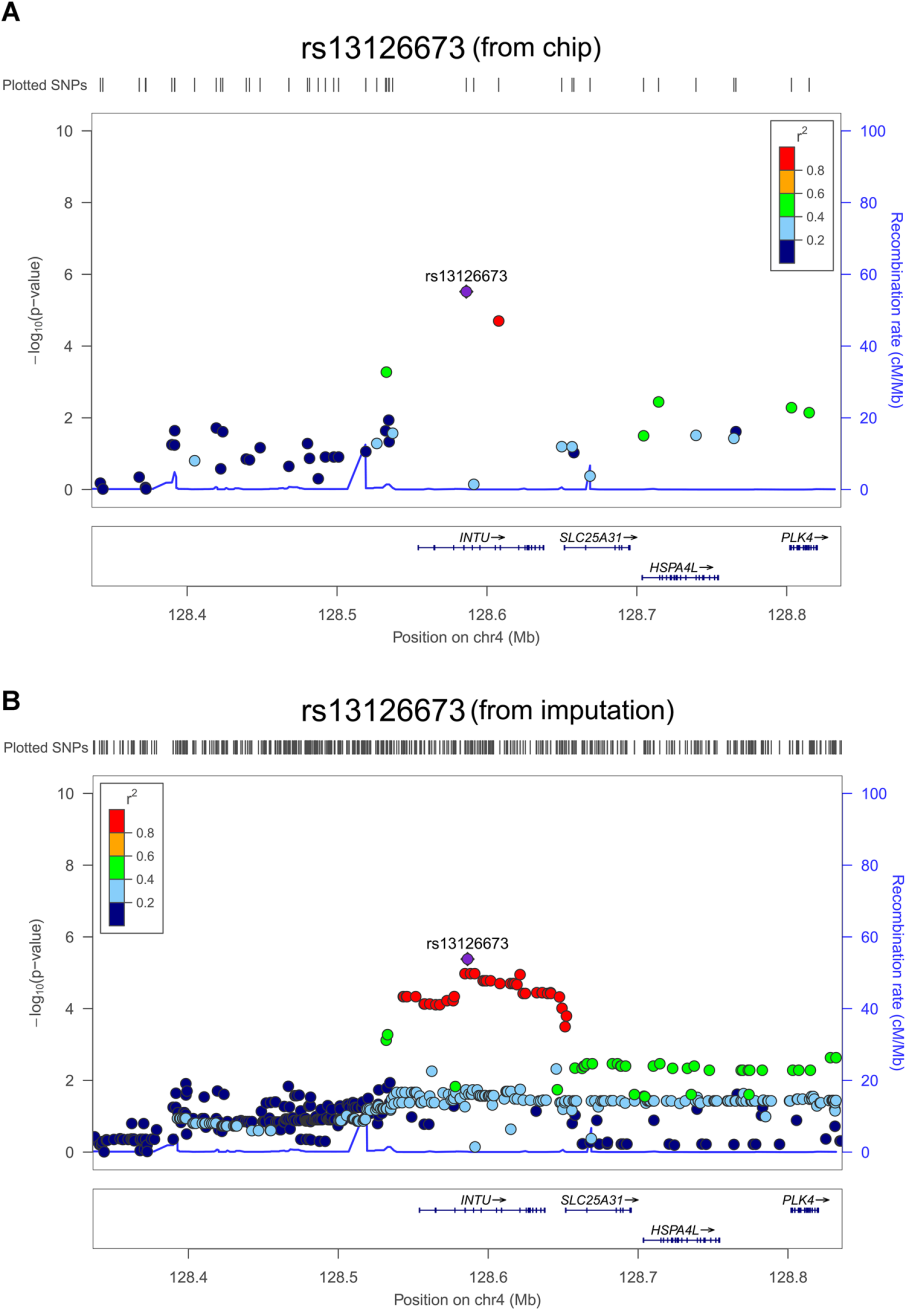
Association plots for the *INTU* locus. (**A**) Regional association plot for the *INTU* locus on chromosome 4 with gene annotation imposed. Each SNP was plotted to represent its chromosomal location (x-axis) and − log10 *P* value (y-axis) for the trend test from the GWAS data. (**B**) After imputation, the SNPs were plotted, and the colors denote the strength of linkage disequilibrium of SNPs to *INTU.*

Of note, to enhance the coverage of SNPs, we imputed all loci using discovery GWAS datasets. After imputation, the top four SNPs included rs10822312 (*P* = 1.08 × 10^−7^) at chromosome 10, rs58991632 (*P* = 1.92 × 10^−6^) and rs2273422 (*P* = 2.42 × 10^−6^) at chromosome 20, and rs12566078 (*P* = 2.50 × 10^−6^) at chromosome 1 were showed in Fig. 4 and Table 4. The whole region of fermitin family member 1 (FERMT1) and INTU were identified using discovery GWAS dataset and imputation results. The reginal association plots were showed in Fig. 5 for FERMT1 and Fig. 6 for INTU.

**Table 4:** Summary of imputation studies of top 21 SNPs in trend tests.

Chr	SNP	Position	Gene	Allele 1	Allele 2	Risk Allele	RAF controls	RAF cases	Imputation Trend P	OR	95% CI	
1	rs12566078	113,847,571	–	A	G	G	0.8333	0.9643	2.50E−06	5.4	2.51	11.62
4	rs13126673	128,586,241	INTU	T	C	C	0.4531	0.6667	4.15E−06	2.414	1.64	3.552
9	rs10733369	2,122,486	SMARCA2	T	C	C	0.4427	0.6548	9.08E−06	2.387	1.624	3.51
9	rs10733553	9,709,405	–	A	G	G	0.6105	0.808	9.38E−06	2.685	1.748	4.123
10	rs10822312	66,314,543	–	T	G	G	0.6042	0.8333	1.80E−07	3.276	2.11	5.085
20	rs58961824	6,084,195	FERMT1	T	A	A	0.4635	0.6746	5.78E−06	2.399	1.629	3.534
20	rs60811855	6,084,782	FERMT1	C	T	T	0.4635	0.6746	5.78E−06	2.399	1.629	3.534
20	rs58790452	6,084,920	FERMT1	G	A	A	0.4635	0.6746	5.78E−06	2.399	1.629	3.534
20	rs61527455	6,085,639	FERMT1	A	G	G	0.4635	0.6746	5.78E−06	2.399	1.629	3.534
20	rs59849941	6,085,718	FERMT1	G	A	A	0.4635	0.6746	5.78E−06	2.399	1.629	3.534
20	rs4407314	6,088,005	FERMT1	A	G	G	0.4635	0.6746	5.78E−06	2.399	1.629	3.534
20	rs16991857	6,088,993	FERMT1	G	A	A	0.4635	0.6746	5.78E−06	2.399	1.629	3.534
20	rs11908294	6,089,741	FERMT1	T	C	C	0.4635	0.6746	5.78E−06	2.399	1.629	3.534
20	rs2326719	6,093,090	FERMT1	A	G	G	0.4635	0.6746	5.78E−06	2.399	1.629	3.534
20	rs4300912	6,093,624	FERMT1	T	C	C	0.4632	0.676	5.85E−06	2.418	1.639	3.569
20	rs4419295	6,094,060	FERMT1	G	A	A	0.4635	0.6746	5.78E−06	2.399	1.629	3.534
20	rs75911461	6,096,872	FERMT1	T	A	A	0.4579	0.6746	3.37E−06	2.454	1.664	3.62
20	rs117103253	6,097,080	FERMT1	T	A	A	0.4579	0.6746	3.37E−06	2.454	1.664	3.62
20	rs58991632	6,098,405	FERMT1	A	G	G	0.4521	0.6746	1.92E−06	2.512	1.701	3.71
20	rs2273422	6,100,241	FERMT1	G	A	A	0.4521	0.6734	2.42E−06	2.498	1.69	3.694
20	rs2273421	6,100,367	FERMT1	C	T	T	0.4521	0.668	4.32E−06	2.438	1.648	3.608

*RAF* risk allele frequency, *Trend P* *P* vale of Trend test, *OR* odds ratio, *CI* confidence interval.

### Genetic regulation of RNA transcription in endometrial tissue with defined genotypes-expression quantitative trait loci (eQTL) analysis

**Figure 7:**
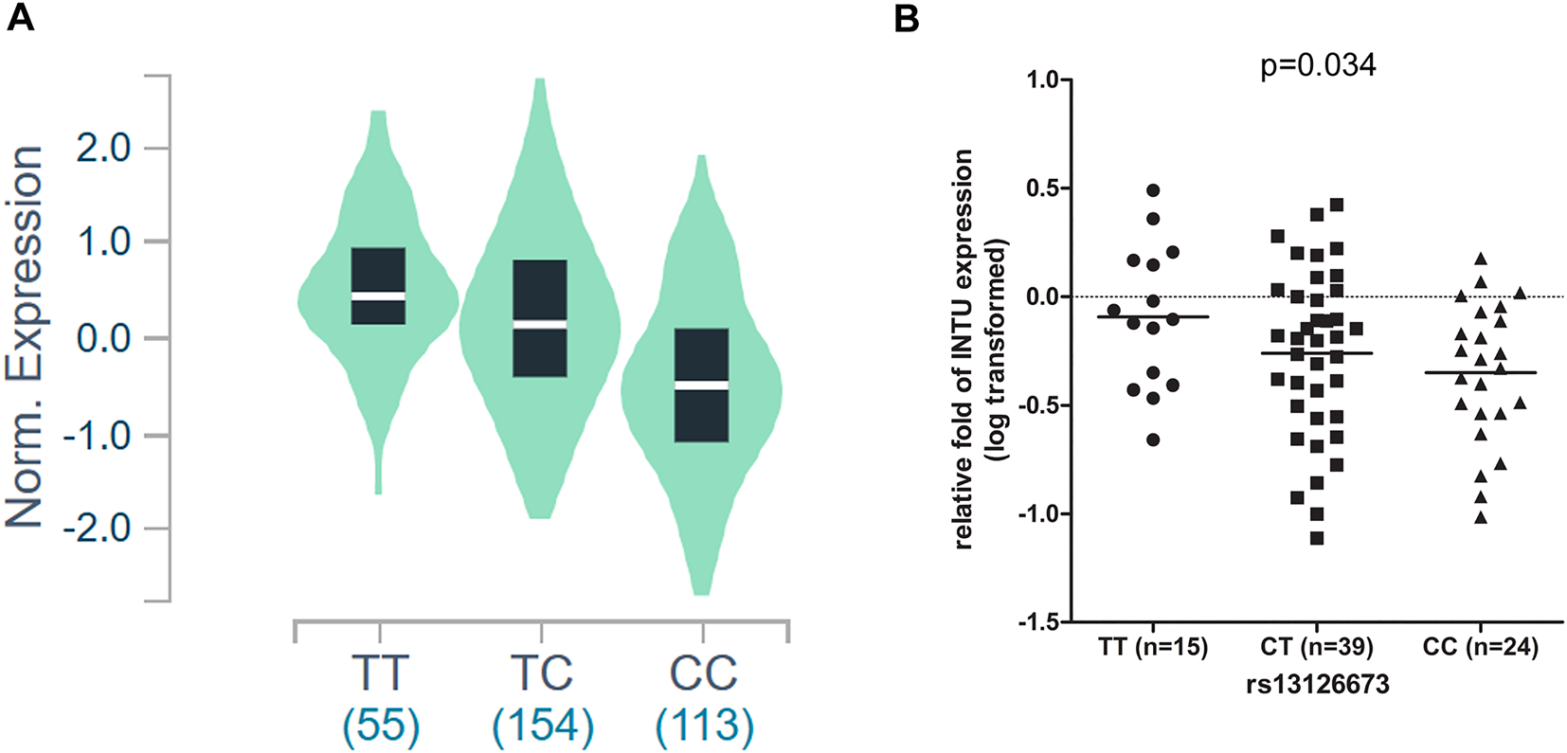
eQTL analysis of the normalized expression level of INTU transcripts. (**A**) SNP rs13126673 was significantly associated with *INTU* expression in the testis (*P* = 5.1 × 10^–33^). Expression data were extracted from GTEx v8, which contained 322 normal subjects. Of note, the individuals with CC genotype had a lower INTU expression level. (**B**) The original GWAS SNP (rs13126673) genotypes [n = 15 (TT), n = 39 (CT), n = 24 (CC)] were determined by Sequenom MassARRAY, and total RNA was extracted from the tissue. The log10 transformed expression level of mRNA for inturned planar cell polarity protein (*INTU*) was measured using reverse transcription quantitative polymerase chain reaction and normalized to that of GAPDH. The horizontal line represents mean and individual sample is represented by dots. After analysis of linear regression, the *P* value was 0.034.

From discovery and replication studies of top 33 SNPs in trend tests, all the *P* values did not reach genome-wide significance. Expression quantitative trait loci (eQTL) explained the variation in expression levels of mRNA. To reveal the eQTL of the top 33 SNPs, we changed the significance threshold to 10^–3^ in joint analysis and found 12 SNPs (Table 3). Among them, only rs13126673 is the putative cis-eQTL. The cis-eQTL analysis was performed to investigate potential association between the variants and expression levels of transcripts. The SNP rs13126673 was located at chromosome 4 in *INTU*. From Genotype-Tissue Expression (GTEx) project database v8, which contained the data of 322 testes samples from normal subjects, and it revealed that individuals with a CC genotype at rs13126673 have lower INTU expression compared with TT carriers, with a *P* value of 5.1 × 10^–33^ (Fig. 7A). To further explore the eQTL in endometriotic tissues, 78 tissue samples from endometriosis patients with recorded SNP genotypes, were used for total RNA extraction and INTU expression was detected using RT-q-PCR. Of note, the C allele of SNP rs13126673 is the risk allele in our GWAS sample (OR = 1.729, 95% CI: 1.309–2.284). Moreover, we detected the expression of INTU of ovarian endometriosis tissue by RT-qPCR in women with endometriosis. Women were categorized as homozygous for the risk allele [n = 24 (CC)], heterozygous for each of the variants [n = 39 (CT)], or homozygous for the alternative allele [n = 15 (TT)]. An eQTL analysis was performed to detect the effects of differing genotypes at SNP rs13126673 on the expression of the INTU transcripts; there was significant association between these genotypes and the expression of INTU transcripts observed (*P* = 0.034) (Fig. 7B).

### Effects of rs13126673 on its RNA secondary structure

**Figure 8:**
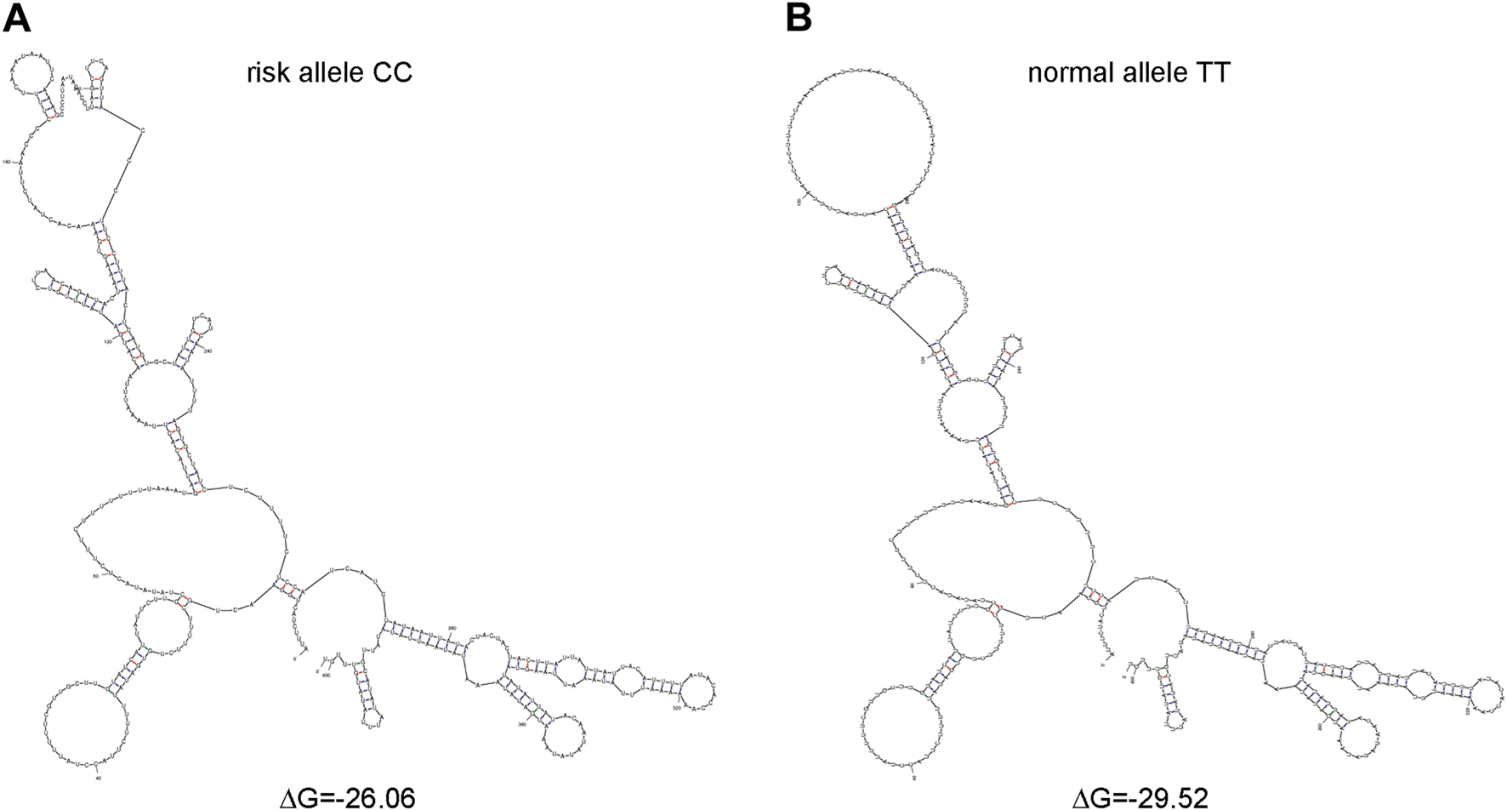
Alteration of computationally predicted RNA secondary structure of the region surrounding rs13126673. The RNA secondary structures in the region at rs13126673 were showed for the (**A**) risk allele CC and (**B**) normal allele TT which were predicted by mfold.

Because rs13126673 is an intronic eQTL, we investigated whether the variation of the SNP influence the RNA secondary structure. Based on the internet-linked computer modeling program mfold^12^, we uncovered that the different predicted RNA secondary structure based on the SNP genotype (Fig. 8A,B). The risk CC genotype had a structure with a ΔG of − 26.06 higher than normal allele TT genotype ΔG of − 29.52, suggesting that TT genotype was more stable.

### Replication of endometriosis-associated single-nucleotide polymorphism from previous genome-wide association studies

Previous studies found that several SNP were associated with endometriosis, including rs2235529 (*P* = 8.65 × 10^–9^), rs7521902 (*P* = 3 × 10^–11^), rs801112 (*P* = 5 × 10^–6^), rs16826658 (*P* = 2 × 10^–6^), rs13394619 (*P* = 6 × 10^–9^), rs1519761 (*P* = 4.7 × 10^–8^), rs6757804 (*P* = 4.05 × 10^–8^) , rs4141819 (*P* = 4.1 × 10^–8^), rs7739264 (*P* = 2.1 × 10^–10^) , rs7798431 (*P* = 1.1 × 10^–7^) , rs12700667 (*P* = 1 × 10^–9^), rs10965235 (*P* = 6 × 10^–12^) , rs1537377 (*P* = 2 × 10^–9^), and rs10859871 (*P* = 5 × 10^–13^)^7–9,13,14^. Thus, we analyzed these SNP by Sequenom MassARRAY and showed the results in Table 5. Among 14 SNPs, only rs13394619 was associated with endometriosis (*P* = 0.01968, OR = 1.416) (Table 5). After multiple test analyses using Bonferroni correction, the association was found to be not significant.

**Table 5:** Replication of endometriosis-associated single-nucleotide polymorphism from previous genome-wide association studies (Endometriosis, n = 241, controls, n = 156).

Chr	Position	SNP	Gene	Allele 1	Allele 2	Risk allele	RAF controls	RAF cases	*P* value	Risk allele OR	95% CI	HWE test P
1	22,123,994	rs2235529	WNT4	C	T	C	0.4375	0.4573	0.6598	1.071	0.8026–1.429	0.4156
1	22,164,231	rs7521902	WNT4	C	A	C	0.4243	0.4411	0.6587	1.078	0.8076–1.44	0.1521
1	228,860,875	rs801112	ROU-ISCAIP2	C	T	C	0.2072	0.2175	0.7224	1.074	0.7566–1.526	0.2363
1	22,159,378	rs16826658	WNT4-ZBTB40	T	G	T	0.4243	0.4553	0.4631	1.125	0.8435–1.502	0.5398
2	11,587,381	rs13394619	GREB1	G	A	G	0.4408	0.5224	0.01968*	1.416	1.062–1.887	0.6933
2	150,776,690	rs1519761	RND3-RBM43	G	A	G	0.3388	0.3760	0.3182	1.17	0.8641–1.585	0.06805
2	150,779,318	rs6757804	RND3-RBM43	C	T	C	0.3849	0.4146	0.5019	1.116	0.8319–1.496	0.8374
2	67,637,543	rs4141819	ETAA1	C	T	C	0.0724	0.0894	0.4297	1.259	0.7387–2.146	1
6	19,785,357	rs7739264	ID4	C	T	T	0.7533	0.7785	0.4851	1.141	0.8124–1.603	0.788
7	25,821,192	rs7798431	NFE2L3/HOXA10	A	G	G	0.5493	0.5976	0.2076	1.213	0.9072–1.621	1
7	25,862,019	rs12700667	NFE2L3/HOXA10	A	G	A	0.1447	0.1829	0.1731	1.323	0.8931–1.96	1
9	22,115,106	rs10965235	CDKN2B-AS1	A	C	C	0.8355	0.8394	0.9212	1.029	0.6987–1.516	0.2936
9	22,169,701	rs1537377	CDKN2B-AS1	C	T	C	0.2566	0.2581	0.9337	1.019	0.7347–1.414	0.6173
12	95,318,100	rs10859871	VEZT	C	A	C	0.2368	0.2459	0.7986	1.057	0.7559–1.477	0.1026

*RAF* risk allele frequency, *Trend P* *P* vale of Trend test, *OR* odds ratio, *CI* confidence interval, *HWE test P* Hardy–Weinberg equilibrium test *P* value of controls.

*indicates *P* < 0.05.

## Discussion

Several novel genetic variants of endometriosis were identified in this study, which, to our knowledge, represents the first report of a GWAS for endometriosis conducted in a Taiwanese population (Figs. 1 and 2). In our two-independent cohort, four novel loci for endometriosis were identified and replicated. Moreover, rs10739199 and rs2025392 were found in linkage disequilibrium (Fig. 3). After GWAS conditional analyses of these two SNPs, the *P* values were increased, and this indicated that they were only both associated. Imputation resulted in more strong signals (Figs. 4, 5, 6). rs13126673 was found that it the cis-eQTL of testes from the GTEx database and in endometriotic tissues (Fig. 7). The rs13126673 may affect the secondary RNA structure (Fig. 8). These results suggest a heritable component in endometriosis and provide new findings into the genetic risk factors of the disease.

We found that the SNPs rs10739199 (*P* = 6.75 × 10^−5^) and rs2025392 (*P* = 8.01 × 10^–5^) located at chromosome 9 in *PTPRD* (protein tyrosine phosphatase, receptor type D) were associated with endometriosis. The other two SNPs were located at chromosome 14 (rs1998998, *P* = 6.5 × 10^−6^) and at chromosome 15 (rs6576560, *P* = 9.7 × 10^−6^). PTPRD is a member of the receptor protein tyrosine phosphatase (PTP) family. Mutations in PTPRD stimulate cell migration and growth in melanoma cell lines, enhance cell proliferation, and abrogate the dephosphorylation of Signal transducer and activator of transcription 3 (STAT3) in human astrocytes^15,16^. Several studies have shown that elevated STAT3 expression occurs in both endometriosis and endometrial cancer, suggesting STAT3 is a potential risk factor for both diseases^17–20^. In the present study, we found two endometriosis-associated SNPs, including rs10739199, and rs2025392 of chromosome 9 within the PTPRD gene. These novel genetic loci may provide new insights into the molecular basis of endometriosis. Moreover, deletions and mutations in PTPRD have been implicated in several tumor types, including endometrioid carcinomas in the Catalogue of Somatic Mutations in Cancer (COSMIC) database, and endometrial cancers^15^. Recently, a cross-disease GWAS meta-analysis revealed the rs2475335 SNP located within PTPRD to be associated with both endometriosis and endometrial cancer^21^. Further study of endometriosis and endometrial cancer models will be important to investigate the underlying biology of diseases-associated variants that increase the risk of both diseases^22^.

The major limitation of this study is the current sample size and it is too low to have real power to detect an association at a genome-wide level. However, endometriosis was reported to be common in patients with other benign gynecological diseases, especially uterine leiomyoma^23^. In the current study, all enrolled women had surgically confirmed diagnoses of endometriosis, and other benign gynecological diseases were used as the control group, hence the sample size was reduced. Recently, a GWAS of uterine myoma identified that eight novel genome-wide significant loci and four loci are also associated with endometriosis risk, suggesting overlapping genetic origins of uterine myoma and endometriosis^24^. In our study, we enrolled women with uterine myoma and other benign gynecological disorders as hospital-based controls rather than population-based controls, which does not inform the strength of endometriosis risk among other gynecological diseases. Because our controls were not healthy women, there may be some potential bias in our study. We also replicate the previous reported 14 SNPs and found that only rs13394619 was associated with endometriosis (*P* = 0.01968), this may due to different controls and populations. After multiple test analyses using Bonferroni correction, the association was found to be not significant. Further GWAS assessing potential susceptibility loci with genome-wide significance in different populations will verify genetic influences in the pathogenesis of endometriosis.

Moreover, we found that rs13126673 was at a novel locus on chromosome 4q28.1 with INTU and was associated with endometriosis. The rs13126673 SNP is an eQTL (expression quantitative trait loci) of INTU, which is a cilia and planar polarity effector with prominent ciliogenic roles in morphogenesis. rs13126673 is an intronic SNPs, which is not located in the predicted regulation regions of INTU transcription based on National Center for Biotechnology Information. Previous studies suggested that SNP may alter RNA/DNA structure and influence gene expression^25,26^. After prediction, the rs13126673 may change secondary RNA conformation (Fig. 8). The role of INTU in the pathogenesis of endometriosis will be worthwhile to study in the future.

In this study, we have provided the first genome-wide evidence in a Taiwanese population of four SNPs located in novel loci that were found to be associated with endometriosis. We have reported novel risk loci for the endometriosis-associated gene, PTPRD, that has been implicated in both endometriosis and endometrial cancer through cross-disease GWAS.

## Methods

### Study subjects

We recruited patients (n = 430) who underwent laparoscopic confirmation and histological analysis of endometriosis (compromising of 126 endometriosis cases from the GWAS and 96 cases from the discovery study) or who had no endometriosis (controls, comprising of 133 controls from the GWAS and 75 controls from the replication study) in the Center for Reproductive Medicine at Taipei Medical University Hospital. All diagnoses were confirmed by pathological analysis and divided into endometriosis and control groups. All women with endometriosis were diagnosed as stage III or IV endometriosis, the indication of control groups for laparoscopy includes myoma, fibrous adhesion, hydrosalpinx, ovarian cysts, teratoma, dermoid cysts, paratubal cysts, epithelial cysts, and simple cysts. A structured questionnaire was used by a trained researcher who interviewed subjects. Informed consent was obtained from each patient, and the study was approved by the Taipei Medical University joint Institutional Review Board (TMU-JIRB 201305035). All participants were Taiwanese women and all methods were carried out in accordance with relevant guidelines and regulations.

### Genotyping and quality control

Genomic DNA was extracted from blood using a DNA whole-blood kit, as per the manufacturer’s instructions (Kurabo Industries, Osaka, Japan). The genotypes of each woman were performed by National Center for Genome Medicine (NCGM) at Academic Sinica using Axiom Genome-Wide TWB (Taiwan Biobank) Array Plate with a total of 653,291 SNPs. The Kinship analysis, quality control of genotypes for each SNP, total call rate, and Hardy–Weinberg Equilibrium (HWE) test were conducted by National Center for Genome Medicine. The HWE test was used to exclude SNPs departed significantly (*P* = 0.0001). After these tests, some SNPs were excluded from further analysis. All sample call rates were > 97%, and the mean individual sample call rate was 99.82 ± 0.37%. The linkage disequilibrium (LD) plot was generated by Haploview version 4.2 (Broad Institute, Cambridge, MA, USA).

### GWAS validation and replication

The top 31 SNPs (*P* < 1 × 10^−4^) from the genome-wide association analysis of 126 endometriosis and 96 non-endometriosis controls were further validated using MALDI-TOP mass spectrometry (MassARRAY, Sequenom) (Supplementary Table 1). SNP genotypes with a success rate over 97% and with over 99% concordance between the two platforms were then genotyped. The rs16911067 and rs16934324 were genotyping by Q-PCR using Taqman SNP genotyping assay and Taqman genotyping master mix (Thermo Fisher Scientific, MA, USA). The previous reported 14 SNPs were replicated using MALDI-TOP mass spectrometry. The multiple comparison correction was analyzed by Bonferroni test using the Graphpad Prism software (California, CA, USA).

### Imputation

To enhance coverage, the untyped SNPs were imputed by IMPUTE2 v2.3 using the 1000 Genome reference panel^27,28^. The National Center for Genome Medicine of Academia Sinica set up the haplotype inferences via the SHAPEIT method for optimizing the imputation rate^29^. We included a 500 kb buffer region on each side of the imputation region for elimination of edge effects and determined the uncertainty of imputed genotypes based on likelihood scoring in SNPTEST v2. Moreover, the frequentist association test of an additive model was used.

### Expression quantitative trait loci (eQTL) analysis

The Genotype-Tissue Expression (GTEx) project database release the summary statistics of eQTL data of SNP rs13126673 for testis (n = 322)^30^. In our study, the eQTL was performed on the total of 78 tissue samples for endometriosis patients with recorded SNP genotypes. The expression of inturned planar cell polarity protein (*INTU*) was detected by reverse transcription quantitative polymerase chain reaction (RT-qPCR) and been described in detail elsewhere^31^. Glyceraldehyde-3-phosphate dehydrogenase (*GAPDH*) mRNA was amplified using q-PCR with the forward primer 5′-gagtccactggcgtcttcac-3′ and reverse primer 5′-gttcacacccatgacgaaca-3′. The *INTU* mRNA was amplified using q-PCR with the forward primer 5′-tcagcgactcgggttcat-3′ and reverse primer 5′-cagccattcaggctcaaga-3′.

### Prediction of RNA secondary structure

The rs13126673 SNP upstream and downstream DNA sequences (approximately 400 bp each) were retrieved by the dbSNP of the National Center for Biotechnology Information. The mfold program was used to predict the RNA structures of retrieved risk allele or normal allele of rs13126673 using default value and calculated the value of ΔG which represented the thermodynamic stability^12^. The smaller ΔG represented the more stable structure^12^.

### Statistical analysis

The statistical method used for GWAS analysis is well-established by the National Center for Genome Medicine^32^. Possible population stratification could affect the association analysis and detection of this was performed using EIGENSTRA2.0. The variance inflation factor for genomic controls was also estimated. GC correction and genome-wide association were performed to compare allele and genotype frequencies between cases and controls using the Cochran–Armitage trend test. The *P* value distribution was showed in a quantile–quantile (Q-Q) plot. Adjustment for principle components suggested that inflation was not due to population stratification. The GWAS conditional analysis was performed as previously described^33^. The analysis of eQTL was performed with linear regression, using IBM SPSS statistics version 22 (New York, USA).

## Supplementary Information

Supplementary Tables.
