# Supplementary material for: Integration of genome-wide association study and expression quantitative trait locus mapping for identification of endometriosis-associated genes
Source: Sci Rep. 2021 Jan 12;11:478. doi: 10.1038/s41598-020-79515-4 (PMC7803948; doi:10.1038/s41598-020-79515-4)
Supplement: Supplementary file 1 — Supplementary Tables. [file 41598_2020_79515_MOESM1_ESM.docx]

**Integration of genome-wide association study and expression quantitative trait locus mapping for identification of endometriosis-associated genes**

Ya-Ching Chou, Ming-Jer Chen, Pi-Hua Chen, Ching-Wen Chang, Mu-Hsien Yu, Yi-Jen Chen, Eing-Mei Tsai, Shih-Feng Tsai, Wun-Syuan Kuo, Chii-Ruey Tzeng *****

| **Supplementary Table 1. Summary of discovery studies of top 33 SNPs in trend tests.** | | | | | | | | | |  |  |  |
| --- | --- | --- | --- | --- | --- | --- | --- | --- | --- | --- | --- | --- |
| Chr. | Position | SNP | Gene | Allele 1 | Allele 2 | Risk Allele | RAF controls | RAF cases | Discovery Trend P | Risk allele OR | 95% CI | |
| 1 | 212387636 | rs12073206 | - | T | C | C | 0.6615 | 0.8214 | 6.41E-05 | 2.354 | 1.517 | 3.654 |
| 2 | 182946950 | rs13395225 | PPP1R1C | A | C | C | 0.6354 | 0.7937 | 8.96E-05 | 2.207 | 1.445 | 3.371 |
| 2 | 202543051 | rs10190770 | MPP4 | T | C | C | 0.5625 | 0.7381 | 7.18E-05 | 2.192 | 1.469 | 3.271 |
| 4 | 128586241 | rs13126673 | INTU | T | C | C | 0.4526 | 0.6707 | 3.03E-06 | 2.463 | 1.667 | 3.639 |
| 5 | 52363759 | rs2056401 | ITGA2 | G | A | A | 0.7292 | 0.8849 | 1.70E-05 | 2.856 | 1.731 | 4.714 |
| 6 | 75160914 | rs9350550 | LOC101928516 | G | A | A | 0.6927 | 0.8532 | 6.77E-05 | 2.578 | 1.62 | 4.101 |
| 7 | 29131494 | rs2192508 | CPVL | G | A | A | 0.4271 | 0.6111 | 9.19E-05 | 2.108 | 1.439 | 3.089 |
| 7 | 90650256 | rs7789771 | CDK14 | G | A | G | 0.02105 | 0.123 | 6.95E-05 | 6.523 | 2.261 | 18.82 |
| 7 | 90660684 | rs1358101 | CDK14 | A | C | A | 0.02083 | 0.127 | 3.84E-05 | 6.836 | 2.374 | 19.68 |
| 7 | 137445794 | rs3778810 | DGKI | A | G | A | 0.2708 | 0.4643 | 5.95E-05 | 2.333 | 1.559 | 3.492 |
| 8 | 83762709 | rs16911067 | - | T | G | G | 0.7158 | 0.8699 | 4.14E-05 | 2.655 | 1.631 | 4.322 |
| 9 | 2121371 | rs10757185 | SMARCA2 | T | G | G | 0.401 | 0.5992 | 2.70E-05 | 2.233 | 1.522 | 3.275 |
| 9 | 2122486 | rs10733369 | SMARCA2 | T | C | C | 0.4427 | 0.6548 | 9.08E-06 | 2.387 | 1.624 | 3.51 |
| 9 | 2130861 | rs6475521 | SMARCA2 | G | C | C | 0.4427 | 0.627 | 8.92E-05 | 2.116 | 1.443 | 3.102 |
| 9 | 9707144 | rs10739199 | PTPRD | G | A | A | 0.6094 | 0.8016 | 1.70E-05 | 2.59 | 1.695 | 3.958 |
| 9 | 9733309 | rs2025392 | PTPRD | T | C | C | 0.8542 | 0.964 | 7.30E-05 | 4.572 | 2.103 | 9.941 |
| 9 | 9734220 | rs2761699 | PTPRD | C | T | T | 0.8594 | 0.9643 | 7.87E-05 | 4.418 | 2.026 | 9.637 |
| 10 | 66314543 | rs10822312 | - | T | G | G | 0.6105 | 0.836 | 2.54E-07 | 3.252 | 2.086 | 5.069 |
| 12 | 22106347 | rs1517283 | - | T | C | C | 0.6042 | 0.784 | 5.55E-05 | 2.378 | 1.566 | 3.611 |
| 12 | 29646146 | rs77182066 | OVCH1 | A | G | A | 0.03125 | 0.144 | 6.17E-05 | 5.215 | 2.149 | 12.65 |
| 12 | 29638052 | rs16934324 | OVCH1/OVH1-AS1 | A | C | A | 0.03125 | 0.1429 | 6.90E-05 | 5.167 | 2.13 | 12.53 |
| 12 | 49627819 | rs12824458 | TUBA1C | G | C | C | 0.5885 | 0.7579 | 9.99E-05 | 2.189 | 1.457 | 3.289 |
| 13 | 97188301 | rs77849897 | HS6ST3 | G | A | A | 0.7234 | 0.8849 | 2.37E-05 | 2.94 | 1.78 | 4.856 |
| 14 | 75785259 | rs10136321 | - | A | T | A | 0.3281 | 0.5317 | 3.79E-05 | 2.325 | 1.575 | 3.434 |
| 14 | 97680819 | rs1998998 | - | A | G | A | 0.1146 | 0.268 | 5.31E-05 | 2.829 | 1.674 | 4.782 |
| 15 | 26577347 | rs6576560 | - | T | C | C | 0.4896 | 0.6905 | 3.09E-05 | 2.326 | 1.576 | 3.432 |
| 16 | 85989898 | rs4843874 | - | C | T | C | 0.01562 | 0.132 | 2.22E-05 | 9.581 | 2.892 | 31.74 |
| 18 | 41362595 | rs1870631 | - | A | G | G | 0.401 | 0.5992 | 3.20E-05 | 2.233 | 1.522 | 3.275 |
| 18 | 67957604 | rs2067687 | SOCS6 | G | C | G | 0.02105 | 0.1468 | 6.78E-06 | 8.002 | 2.8 | 22.87 |
| 18 | 68041124 | rs2251133 | - | C | T | C | 0.03646 | 0.1548 | 2.72E-05 | 4.839 | 2.114 | 11.08 |
| 20 | 6059674 | rs2236148 | FERMT1 | C | A | C | 0.3594 | 0.5595 | 5.58E-05 | 2.264 | 1.54 | 3.33 |
| 20 | 6074873 | rs2232071 | FERMT1 | C | T | T | 0.4635 | 0.664 | 1.78E-05 | 2.287 | 1.554 | 3.366 |
| 20 | 6093090 | rs2326719 | FERMT1 | A | G | G | 0.4684 | 0.6746 | 9.19E-06 | 2.353 | 1.596 | 3.468 |

RAF: risk allele frequency, Trend P: *P* vale of Trend test, OR: odds ratio, CI: confidence interval

| **Supplementary Table 2. Summary of replication studies of top 33 SNPs in trend tests.** | | | | | | | | | |  |  |  |
| --- | --- | --- | --- | --- | --- | --- | --- | --- | --- | --- | --- | --- |
| Chr. | Position | SNP | Gene | Allele1 | Allele 2 | Risk Allele | RAF controls | RAF cases | Replication Trend P | Risk allele OR | 95% CI | |
| 1 | 212387636 | rs12073206 | - | T | C | C | 0.6622 | 0.697 | 4.73E-01 | 1.173 | 0.7633 | 1.804 |
| 2 | 182946950 | rs13395225 | PPP1R1C | A | C | C | 0.7365 | 0.7115 | 5.98E-01 | 0.8826 | 0.5607 | 1.389 |
| 2 | 202543051 | rs10190770 | MPP4 | T | C | C | 0.7027 | 0.6705 | 4.86E-01 | 0.8607 | 0.5566 | 1.331 |
| 4 | 128586241 | rs13126673 | INTU | T | C | C | 0.5135 | 0.5611 | 3.64E-01 | 1.211 | 0.8083 | 1.814 |
| 5 | 52363759 | rs2056401 | ITGA2 | G | A | A | 0.8311 | 0.8447 | 7.16E-01 | 1.105 | 0.6417 | 1.904 |
| 6 | 75160914 | rs9350550 | LOC101928516 | G | A | A | 0.7397 | 0.7576 | 6.74E-01 | 1.1 | 0.6909 | 1.75 |
| 7 | 29131494 | rs2192508 | CPVL | G | A | A | 0.5946 | 0.5568 | 4.43E-01 | 0.8566 | 0.5695 | 1.289 |
| 7 | 90650256 | rs7789771 | CDK14 | G | A | G | 0.08108 | 0.1077 | 3.98E-01 | 1.368 | 0.6734 | 2.778 |
| 7 | 90660684 | rs1358101 | CDK14 | A | C | A | 0.08108 | 0.1031 | 4.80E-01 | 1.302 | 0.6389 | 2.654 |
| 7 | 137445794 | rs3778810 | DGKI | A | G | A | 0.3514 | 0.3577 | 9.00E-01 | 1.028 | 0.6741 | 1.568 |
| 8 | 83762709 | rs16911067 | - | T | G | G | 0.8592 | 0.8071 | 1.82E-01 | 0.6858 | 0.3893 | 1.208 |
| 9 | 2121371 | rs10757185 | SMARCA2 | T | G | G | 0.5946 | 0.5267 | 1.92E-01 | 0.7588 | 0.5046 | 1.141 |
| 9 | 2122486 | rs10733369 | SMARCA2 | T | C | C | 0.6216 | 0.5687 | 3.01E-01 | 0.8026 | 0.5313 | 1.212 |
| 9 | 2130861 | rs6475521 | SMARCA2 | G | C | C | 0.6149 | 0.5534 | 2.36E-01 | 0.7763 | 0.5147 | 1.171 |
| 9 | 9707144 | rs10739199 | PTPRD | G | A | A | 0.6486 | 0.7154 | 1.73E-01 | 1.361 | 0.8841 | 2.097 |
| 9 | 9733309 | rs2025392 | PTPRD | T | C | C | 0.9189 | 0.947 | 2.45E-01 | 1.576 | 0.7088 | 3.503 |
| 9 | 9734220 | rs2761699 | PTPRD | C | T | T | 0.9189 | 0.9385 | 4.54E-01 | 1.346 | 0.6185 | 2.927 |
| 10 | 66314543 | rs10822312 | - | T | G | G | 0.7162 | 0.7121 | 9.34E-01 | 0.9801 | 0.6276 | 1.531 |
| 12 | 22106347 | rs1517283 | - | T | C | C | 0.6689 | 0.6742 | 9.12E-01 | 1.024 | 0.6675 | 1.572 |
| 12 | 29646146 | rs77182066 | OVCH1 | A | G | A | 0.1284 | 0.1069 | 5.04E-01 | 0.8124 | 0.4366 | 1.512 |
| 12 | 29638052 | rs16934324 | OVCH1/OVH1-AS1 | A | C | A | 0.1338 | 0.1063 | 4.05E-01 | 0.77 | 0.4115 | 1.441 |
| 12 | 49627819 | rs12824458 | TUBA1C | G | C | C | 0.7162 | 0.6885 | 5.24E-01 | 0.8756 | 0.562 | 1.364 |
| 13 | 97188301 | rs77849897 | HS6ST3 | G | A | A | 0.8493 | 0.8168 | 3.95E-01 | 0.791 | 0.4559 | 1.372 |
| 14 | 75785259 | rs10136321 | - | T | A | A | 0.5608 | 0.5577 | 9.48E-01 | 0.9874 | 0.6576 | 1.483 |
| 14 | 97680819 | rs1998998 | - | A | G | A | 0.1486 | 0.2386 | 2.38E-02 | 1.795 | 1.052 | 3.062 |
| 15 | 26577347 | rs6576560 | - | T | C | C | 0.527 | 0.6364 | 3.78E-02 | 1.571 | 1.044 | 2.363 |
| 16 | 85989898 | rs4843874 | - | C | T | C | 0.1081 | 0.09091 | 5.61E-01 | 0.825 | 0.4233 | 1.608 |
| 18 | 41362595 | rs1870631 | - | A | G | G | 0.5676 | 0.5758 | 8.66E-01 | 1.034 | 0.6886 | 1.553 |
| 18 | 67957604 | rs2067687 | SOCS6 | G | C | G | 0.1507 | 0.09542 | 8.81E-02 | 0.5946 | 0.3222 | 1.097 |
| 18 | 68041124 | rs2251133 | - | C | T | C | 0.2095 | 0.1385 | 6.51E-02 | 0.6066 | 0.3571 | 1.03 |
| 20 | 6059674 | rs2236148 | FERMT1 | C | A | C | 0.4662 | 0.4237 | 4.11E-01 | 0.8416 | 0.5611 | 1.262 |
| 20 | 6074873 | rs2232071 | FERMT1 | C | T | T | 0.5068 | 0.4886 | 7.28E-01 | 0.9301 | 0.6218 | 1.391 |
| 20 | 6093090 | rs2326719 | FERMT1 | A | G | G | 0.527 | 0.4885 | 4.56E-01 | 0.857 | 0.5721 | 1.284 |

RAF: risk allele frequency, Trend P: *P* vale of Trend test, OR: odds ratio, CI: confidence interval

| **Supplementary Table 3. Summary of joint studies of top 33 SNPs in trend tests.** | | | | | | | | | |  |  |  |
| --- | --- | --- | --- | --- | --- | --- | --- | --- | --- | --- | --- | --- |
| Chr. | Position | SNP | Gene | Allele1 | Allele 2 | Risk Allele | RAF controls | RAF cases | Joint  Trend P | Risk allele OR | 95% CI | |
| 1 | 212387636 | rs12073206 | - | T | C | C | 0.6618 | 0.7578 | 2.14E-03 | 1.599 | 1.182 | 2.162 |
| 2 | 182946950 | rs13395225 | PPP1R1C | A | C | C | 0.6794 | 0.752 | 1.86E-02 | 1.43 | 1.056 | 1.938 |
| 2 | 202543051 | rs10190770 | MPP4 | T | C | C | 0.6235 | 0.7035 | 1.22E-02 | 1.432 | 1.072 | 1.913 |
| 4 | 128586241 | rs13126673 | INTU | T | C | C | 0.4793 | 0.6142 | 1.10E-04 | 1.729 | 1.309 | 2.284 |
| 5 | 52363759 | rs2056401 | ITGA2 | G | A | A | 0.7735 | 0.8643 | 4.76E-04 | 1.865 | 1.304 | 2.668 |
| 6 | 75160914 | rs9350550 | LOC101928516 | G | A | A | 0.713 | 0.8043 | 1.75E-03 | 1.654 | 1.2 | 2.279 |
| 7 | 29131494 | rs2192508 | CPVL | G | A | A | 0.5 | 0.5833 | 1.42E-02 | 1.4 | 1.063 | 1.844 |
| 7 | 90650256 | rs7789771 | CDK14 | G | A | G | 0.04734 | 0.1152 | 7.21E-04 | 2.621 | 1.481 | 4.638 |
| 7 | 90660684 | rs1358101 | CDK14 | A | C | A | 0.04706 | 0.1148 | 7.03E-04 | 2.626 | 1.484 | 4.646 |
| 7 | 137445794 | rs3778810 | DGKI | A | G | A | 0.3059 | 0.4102 | 2.72E-03 | 1.578 | 1.18 | 2.109 |
| 8 | 83762709 | rs16911067 | - | T | G | G | 0.7771 | 0.838 | 2.43E-02 | 1.484 | 1.044 | 2.108 |
| 9 | 2121371 | rs10757185 | SMARCA2 | T | G | G | 0.4853 | 0.5623 | 2.76E-02 | 1.362 | 1.035 | 1.793 |
| 9 | 2122486 | rs10733369 | SMARCA2 | T | C | C | 0.5206 | 0.6109 | 9.59E-03 | 1.446 | 1.096 | 1.907 |
| 9 | 2130861 | rs6475521 | SMARCA2 | G | C | C | 0.5176 | 0.5895 | 3.90E-02 | 1.338 | 1.015 | 1.763 |
| 9 | 9707144 | rs10739199 | PTPRD | G | A | A | 0.6265 | 0.7578 | 6.75E-05 | 1.866 | 1.384 | 2.515 |
| 9 | 9733309 | rs2025392 | PTPRD | T | C | C | 0.8824 | 0.9553 | 8.01E-05 | 2.846 | 1.671 | 4.848 |
| 9 | 9734220 | rs2761699 | PTPRD | C | T | T | 0.8853 | 0.9512 | 4.05E-04 | 2.524 | 1.497 | 4.255 |
| 10 | 66314543 | rs10822312 | - | T | G | G | 0.6568 | 0.7724 | 4.36E-04 | 1.773 | 1.307 | 2.405 |
| 12 | 22106347 | rs1517283 | - | T | C | C | 0.6324 | 0.7276 | 3.54E-03 | 1.553 | 1.158 | 2.084 |
| 12 | 29646146 | rs77182066 | OVCH1 | A | G | A | 0.07353 | 0.125 | 1.53E-02 | 1.8 | 1.109 | 2.921 |
| 12 | 29638052 | rs16934324 | OVCH1/OVH1-AS1 | A | C | A | 0.07485 | 0.1245 | 2.05E-02 | 1.758 | 1.082 | 2.856 |
| 12 | 49627819 | rs12824458 | TUBA1C | G | C | C | 0.6441 | 0.7227 | 1.07E-02 | 1.44 | 1.072 | 1.933 |
| 13 | 97188301 | rs77849897 | HS6ST3 | G | A | A | 0.7784 | 0.8502 | 7.89E-03 | 1.615 | 1.134 | 2.302 |
| 14 | 75785259 | rs10136321 | - | T | A | A | 0.4294 | 0.5449 | 9.59E-04 | 1.591 | 1.207 | 2.098 |
| 14 | 97680819 | rs1998998 | - | A | G | A | 0.1294 | 0.2529 | 6.50E-06 | 2.277 | 1.567 | 3.31 |
| 15 | 26577347 | rs6576560 | - | T | C | C | 0.5059 | 0.6628 | 9.70E-06 | 1.92 | 1.451 | 2.541 |
| 16 | 85989898 | rs4843874 | - | C | T | C | 0.05588 | 0.1109 | 6.15E-03 | 2.107 | 1.23 | 3.61 |
| 18 | 41362595 | rs1870631 | - | A | G | G | 0.4735 | 0.5872 | 8.31E-04 | 1.582 | 1.2 | 2.084 |
| 18 | 67957604 | rs2067687 | SOCS6 | G | C | G | 0.07738 | 0.1206 | 4.16E-02 | 1.635 | 1.012 | 2.644 |
| 18 | 68041124 | rs2251133 | - | C | T | C | 0.1118 | 0.1465 | 1.41E-01 | 1.364 | 0.899 | 2.07 |
| 20 | 6059674 | rs2236148 | FERMT1 | C | A | C | 0.4059 | 0.4903 | 1.84E-02 | 1.408 | 1.067 | 1.857 |
| 20 | 6074873 | rs2232071 | FERMT1 | C | T | T | 0.4824 | 0.5739 | 8.81E-03 | 1.446 | 1.098 | 1.904 |
| 20 | 6093090 | rs2326719 | FERMT1 | A | G | G | 0.4941 | 0.5801 | 1.34E-02 | 1.414 | 1.073 | 1.864 |

RAF: risk allele frequency, Trend P: *P* vale of Trend test, OR: odds ratio, CI: confidence interval
